# Is Arc mRNA Unique: A Search for mRNAs That Localize to the Distal Dendrites of Dentate Gyrus Granule Cells Following Neural Activity

**DOI:** 10.3389/fnmol.2017.00314

**Published:** 2017-10-10

**Authors:** Christopher A. de Solis, Anna A. Morales, Matthew P. Hosek, Alex C. Partin, Jonathan E. Ploski

**Affiliations:** ^1^School of Behavioral and Brain Sciences and the Department of Molecular & Cell Biology, University of Texas at Dallas, Richardson, TX, United States; ^2^UT Southwestern Medical Center, Dallas, TX, United States

**Keywords:** Arc, MiR132, RNA transport, dendrites, hippocampus, perforant pathway, dentate gyrus

## Abstract

There have been several attempts to identify which RNAs are localized to dendrites; however, no study has determined which RNAs localize to the dendrites following the induction of synaptic activity. We sought to identify all RNA transcripts that localize to the distal dendrites of dentate gyrus granule cells following unilateral high frequency stimulation of the perforant pathway (pp-HFS) using Sprague Dawley rats. We then utilized laser microdissection (LMD) to very accurately dissect out the distal 2/3rds of the molecular layer (ML), which contains these dendrites, without contamination from the granule cell layer, 2 and 4 h post pp-HFS. Next, we purified and amplified RNA from the ML and performed an unbiased screen for 27,000 RNA transcripts using Affymetrix microarrays. We determined that Activity Regulated Cytoskeletal Protein (Arc/Arg3.1) mRNA, exhibited the greatest fold increase in the ML at both timepoints (2 and 4 h). In total, we identified 31 transcripts that increased their levels within the ML following pp-HFS across the two timepoints. Of particular interest is that one of these identified transcripts was an unprocessed micro-RNA (pri-miR132). Fluorescent *in situ* hybridization and qRT-PCR were used to confirm some of these candidate transcripts. Our data indicate Arc is a unique activity dependent gene, due to the magnitude that its activity dependent transcript localizes to the dendrites. Our study determined other activity dependent transcripts likely localize to the dendrites following neural activity, but do so with lower efficiency compared to Arc.

## Introduction

Changes that lead to lasting alterations in synaptic strength are believed to require *de novo* protein synthesis (Stanton and Sarvey, 1984; Deadwyler et al., 1987). This observation raises an intriguing question: how does one nucleus that globally controls transcription for each individual neuron effectively subserve 1000s of synapses that can be modified in a spatially and temporally restricted manner? Accumulating evidence suggests one possibility is to target specific mRNAs to dendritic spines, where these mRNAs can be locally translated in a spatially and temporally restricted manner following synaptic activity, leading to synapse specific modifications (Crino and Eberwine, 1996; Tiedge and Brosius, 1996; Weiler et al., 1997; Steward et al., 1998; Huber et al., 2000; Kacharmina et al., 2000). Currently, there is strong support for this model, and numerous studies have reported the existence of mRNAs within dendrites (Miyashiro et al., 1994; Tian et al., 1999; Eberwine et al., 2002; Sung et al., 2004; Poon et al., 2006; Zhong et al., 2006; Cajigas et al., 2012). These studies vary on the methodology utilized to detect dendritically localized mRNAs and the number of mRNAs detected, with some studies only identifying 10s of mRNAs whereas others identify 1000s. These previous studies; however, were not designed to identify transcripts which localize to dendrites following synaptic activity.

Currently, only a few transcripts have been identified to be dendritically localized following synaptic activity, all of which were identified serendipitously (Link et al., 1995; Lyford et al., 1995; Tongiorgi et al., 1997; Mori et al., 2000; Havik et al., 2003; Tiruchinapalli et al., 2003). The most note-worthy transcript to exhibit this is the Activity Regulated Cytoskeletal protein (Arc/Arg3.1) mRNA. Arc mRNA is undoubtedly, thus far, the one RNA that exhibits the most convincing and robust localization to dendrites following neural activity and this has been elegantly, and unambiguously, demonstrated *in vivo* within the hippocampus following high frequency stimulation of the perforant path (pp-HFS) (Link et al., 1995; Lyford et al., 1995; Farris et al., 2014). Brain derived neurotrophic factor (BDNF) is another mRNA thought to be transported to the dendrites following neural activity; however, newer data has questioned this notion (Will et al., 2013). This potential reversal is, in part, due to the difficulty of reliably detecting dendritically localized mRNAs.

Over the last decade it has become apparent that there are 100s of genes that are induced transcriptionally following synaptic activity (i.e., neural activity dependent genes) (Lin et al., 2008; Ploski et al., 2010). It currently remains unknown how many of these genes might have their mRNAs transported to dendrites following synaptic activity. Because some activity-induced transcripts transported to the dendrites may degrade quickly, it is necessary to design an experiment to identify which activity-dependent transcripts localize to the dendrites following synaptic activity (Will et al., 2013). For this reason it is not surprising that most of the aforementioned studies did not identify Arc in their screens (Miyashiro et al., 1994; Tian et al., 1999; Eberwine et al., 2002; Sung et al., 2004; Zhong et al., 2006), and this is likely, in part, due to the limited time Arc resides within this compartment. Therefore, we sought to determine which activity dependent genes have their RNAs transported to the dendrites following neural activity.

To this end, we developed a unique method to identify transcripts that are transported to distal dendrites following synaptic activity using a combination of *in viv*o induction of long term potentiation (LTP) within the rat dentate gyrus followed by laser microdissection of the dentate gyrus molecular layer, and whole genome gene expression analysis.

## Materials and Methods

### Subjects

Adult male Sprague Dawley rats (Charles Rivers Laboratories) weighing 300–350 gm were housed in pairs in plastic cages and maintained on a 12 h light/dark cycle. Food and water were provided *ad libitum* throughout the experiment. Animal use procedures were in strict accordance with the National Institutes of Health Guide for the Care and Use of Laboratory Animals and were approved by the University of Texas at Dallas Animal Care and Use Committee.

### Electrical Stimulation Experiments

Electrical stimulation experiments were performed as previously described (Ploski et al., 2008, 2010). For LTP stimulation experiments, Sprague Dawely rats (300–350 g) were anesthetized with urethane (2 i.p. injections at 10 min intervals; total of 1.6 mg/kg) and placed in a stereotaxic frame. The skull was exposed and the rats were implanted with a concentric bipolar stimulating electrode (Kopf Instruments model #NEX-100) into the angular bundle of the perforant path (-7.8 AP, 4 ML, -3.4 DV). One-half hour following implantation of the stimulating electrode, rats were given LTP-inducing HFS, which consisted of six trains of pulses (400 Hz, 20 ms/pulse), delivered at a 10 s interval and repeated six times at an interval of 2 min with a stimulation intensity of 500 μAmps, 100 μs. This protocol has been widely used in the perforant-dentate pathway to produce reliable and robust potentiation of perforant path synapses (Davis et al., 2000; Messaoudi et al., 2007; Ploski et al., 2010). In all stimulation experiments current was applied such that it traveled from the tip to the tube of the bipolar stimulation electrode. The rats were sacrificed at either 2 or 4 h following HFS and the brain was dissected and immediately frozen on powdered dry ice and stored at -80°C until further processing.

### Immunohistochemistry of Arc for Confirmation of Electrical Stimulation of the Perforant Pathway

At the appropriate time point, the brains were rapidly dissected and promptly frozen with powdered dry ice and stored at -80°C until further processing. Twenty-micron coronal sections containing the anterior dorsal hippocampus were obtained and rapidly frozen immediately after being mounted on Fisher Superfrost slides. Sections were fixed in 4% PFA in PB Buffer (NaH_2_PO_4_ monohydrate 125 mM, NaOH 96 mM) for 10 min. Sections were then washed 3x for 10 min in PBS-A (NaCl 150 mM, NaOH 96 mM, NaH_2_PO_4_ 125 mM), followed by a 1 h incubation in blocking solution 1% bovine serum albumin (1% BSA, Sigma, Cat #A-3059; 0.1% Triton X-100, AmericanBio, #AB02025), slices were incubated overnight at room temperature in anti-Arc/Arg3.1 antibody (1:500; Santa Cruz, #17839) in blocking solution, at room temperature in a humid chamber. On the following day sections were washed 3x with PBS-B (NaCl 150 mM, NaOH 10 mM, NaH_2_PO_4_ 12.5 mM) followed by a 2 h incubation in Texas Red secondary antibody (1:1000, Life Technologies, #T6390). Sections were then washed 3x with PBS-B and mounted (VectaShield, H-1000). IHC images were captured at 100× magnification using an Olympus BX51 upright fluorescence microscope with an Olympus DP71 Digital Camera and DP manager software. Only samples exhibiting robust and uniform expression of Arc in the granule and molecular layers for the stimulated side of the brain were selected for further study. We attempted to deliver pp-HFS to 43 animals of which we found that 23 exhibited robust Arc IHC signal within the dentate gyrus and these were the animals used for further study.

### Laser Microdissection and RNA Purification

Dissection and purification procedures were performed as previously described in Partin et al. (2013). Ten-micron sections containing the dorsal hippocampus were taken and placed onto MMI Laser Microdissection (LMD) slides (MMI, #50102) and stored at -80°C until further processing. Immediately prior to microdissection, the slides were subjected to ethanol dehydration (75% 30 s, 95% 30 s, 100% 30 s, xylene 30 s, xylene 5 min) using Histogen LCM Frozen Section Staining Kit (Arcturus, Mountain View, CA, United States). Microdissection was performed via laser microdissection on a SmartCut Laser Microdissection System, which was mounted on an Olympus CKX41 inverted microscope. For samples that were used in microarray analysis, 120 sections were dissected per sample (control, *n* = 3; 2 h, *n* = 3; 4 h, *n* = 3) from the anterior-medial dentate gyrus, in which the distal 2/3rds of the molecular layer was collected. For samples utilized in qRT-PCR analysis, another set of animals were stimulated, dissected and subjected to qRT-PCR analysis. These animals had the distal 1/3rd of the molecular layer collected, as well as the granule layer across twenty-one 10 micron sections per sample (control, *n* = 5; 2 h, *n* = 4; 4 h, *n* = 4). Control samples consisted of the contralateral, non-stimulated molecular and granule layers, respectively. Samples were collected in 25 μL of cell lysis buffer and purified using RNAqueos-Micro Kit (Ambion, #AM1931). Samples were re-purified via precipitation using Pellet Paint NF (Novagen) and resuspended in a 10 μL volume. All steps were carried out according to manufacturers’ instructions. Purified RNA was converted to cDNA (SuperScript ll; Invitrogen, #18064014) for qRT-PCR analysis or amplified for microarray analysis.

### RNA Amplification

RNA in a 10 μL volume was amplified using MessageAmp II aRNA Amplification Kit (Ambion) according to the manufacturer’s instructions. The amplified RNA from this first round of RNA amplification was subjected to a second round of RNA amplification using MessageAmp II-Biotin Enhanced aRNA Amplification Kit and was performed according to the manufacturer’s instructions to yield Biotin labeled aRNA suitable for Affymetrix microarray analysis. RNA from second round amplification was also converted to cDNA for qRT-PCR analysis.

### DNA Microarray

Was essentially performed as previously described (Ploski et al., 2006, 2010; Partin et al., 2013). A total of 9 microarray hybridizations were performed at the UTSW microarray facility using Affymetrix single channel Rat Genome 230 2.0 Arrays, *n* = 3 per group (3x unstimulated, 3 × 2 h post-stimulation and 3 × 4 h post-stimulation). Gene lists were created based on the relatively stringent criteria that the gene must exhibit an average fold difference of threefold or greater in pair wise comparisons between the unstimulated and stimulated samples, with a *t*-test *p*-value of *p* < 0.05. Importantly, the Microarray Quality Control (MAQC) Consortium has reported that this approach can be successful in identifying reproducible gene lists (Shi et al., 2006). Supplemental data including *p*-values, gene bank accession numbers and full gene names, etc. are included in Supplementary File S1.

### Quantitative Real-Time PCR (qRT-PCR)

Quantitative RT-PCR was performed using the ΔΔ*C*_t_ method as we have described previously (Ploski et al., 2006, 2008, 2010; de Solis et al., 2015) using a CFX96 Real-Time System thermocycler (BioRad, #1845096) and QuantiTect SYBR Green PCR Kit (Qiagen, #204143) with custom designed primers at a concentration of 300 nM. For qRT-PCR experiments, all samples were performed in duplicate and relative gene concentrations were normalized against GAPDH levels. qRT-PCR was performed with the following conditions [(95°C for 15 min) ((94°C for 30 s, 55°C for 30 s, 72°C for 30 s) × (35 cycles))] in a standard 20 μL reaction. PCR primers utilized for qRT-PCR were examined for their efficiency of PCR amplification and were found to be ∼99% efficient. Primer sequences can be found in Supplementary File S2.

### PCR Amplification of the RM2 Locus

Standard PCR was performed using Titanium Taq (Clontech Laboratories, #639208) in a 20 μL volume from rat cDNA from the molecular layer using the following conditions [(95°C for 60 s) ((95°C for 30 s, 68°C for 60 s, 72°C for 30 s) × (35 cycles))].

PCR reactions were performed with the following primer sets: (miR-212 FP TAACAGTCTCCAGTCACGGCCACCGACGCC; RM2 RP GGTCTCACTGTAGTTCTGGCTAGCCTTGAACTCACAGAAACCC) (miR-132 FP CAGGGCAACCGTGGCTTTCGATTGTTACTGTGGGAACCGG; RM2 RP). PCR products of 1.5 kb and ∼0.6 kb were obtained for the PCR amplification using the miR-132 FP and RM2 RP primers. No PCR products were obtained using the miR-212 FP and RM2 RP. The PCR products were cloned into the pCR4-Topo vector via a TA cloning kit (Invitrogen, #450030) and sequenced (Retrogen, Inc.).

### RNA *in Situ* Probe Production

The probe template for Arc was a generous gift from Oswald Steward and it was designed to target the entire Arc coding region (Guzowski et al., 1999; Farris et al., 2014). The probe template for Homer1a was designed to target the Homer1a 3′ untranslated region and was a generous gift from John Guzowski (Guzowski et al., 1999; Vazdarjanova et al., 2002). The DNA templates to produce fluorescent *in situ* hybridization (FISH) RNA probes for Egr3, Egr4, Sox11, Ptgs2, pri-miR132 were PCR amplified from rat cDNA and these PCR products were cloned into the pCR4-TOPO vector using a TA cloning kit (Invitrogen, #450030). FISH Probes were prepared using MAXIscript T7/T3 Transcription Kit (Ambion, #AM1324) and were labeled with UTP-Digoxigenin (Roche, #11209256910). These probes were purified using mini Quick Spin RNA Columns (Roche, #11814427001). RNA probes that were labeled with radioactivity was performed as previously described (Ploski et al., 2010; Partin et al., 2013). DNA templates for Arc and Nurr1 were PCR amplified and used to produce a radiolabeled probe using a T7-based *in vitro* transcription kit (Megashortscript; Ambion) using [^35^S]CTP (1.5 μCi) (PerkinElmer). Removal of unincorporated nucleotides after the *in vitro* transcription reaction was performed using sepharose spin columns (Roche). PCR primers used to generate DNA templates for RNA probe production are listed in Supplementary File S2.

### *In Situ* Hybridization and Fluorescent *in Situ* Hybridization (FISH)

*In situ hybridization* was performed as previously described (Ploski et al., 2010; Partin et al., 2013). FISH was performed as previously described (Farris et al., 2014). For FISH, Prior to hybridization, sections were fixed in 4% PFA in PBS (pH 7.4) for 5 min, rinsed in 2x SSC (AmericanBio, #AB13156) for 2 min, incubated in 0.50% acetic anhydride (Sigma, #320102) in 1.5% triethanolamine (Sigma, #90276) for 10 min and then treated with 1:1 acetone (Sigma, #270725): methanol (Fisher, #A412) for 5 min. Prehybridization was performed at 56°C for 1 h in hybridization buffer [(50% formamide (AmericanBio, #AB00600), 5x SSC, 1.25x denhardt’s solution (AmericanBio, #AB03075), 250 μg/mL, *E. coli* tRNA (Sigma, #R1753), 500 μg/mL salmon sperm (Sigma, #D7656) and 5% dextran sulfate (AmericanBio, # AB00426)]. Sections were hybridized overnight (12–14 h) in hybridization buffer containing 100 ng of probe. Post-hybridization washes (1 × 2x SSC 5 min, 1 × 2x SSC 10 min) were followed by treatment with RNAse (10 μg/mL; Fisher, #BP2539) for 15 min at 37°C. Following 2 min × 5 min washes in 2x SSC, sections were placed in 0.5x SSC for 10 min, followed by a 30 min incubation in 0.5x SSC at 56°C. After 2 min × 5 min washes at RT in 0.5x SSC, sections were incubated in 2x SSC containing 3% hydrogen peroxide to inhibit endogenous peroxidases. Sections were then washed 2 × 2x SSC for 5 min before being placed into TBS (0.01 M Tris-HCL, 0.1 M NaCl, pH 7.5) for 5 min. Sections were blocked with 2% blocking buffer (Roche, #11096176001) in TBS, containing 5% goat serum for 30 min, followed by a 2 h incubation in anti-DIG-HRP (1:200; Perkin Elmer, #NEF832001EA) in blocking buffer. Sections were then washed 3x in TBS-T (TBS containing 0.05% Tween 20, pH 7.5) for 5 min, followed by a 30 min incubation with Cy3 in 1x amplification buffer (1:50; TSA Plus Cyanine 3 System, Perkin Elmer, #NEL744001KT). After 3x wash in TBS-T for 5 min each, slides were then coverslipped with Vectashield HardSet Mounting Medium (Vector Laboratories). Images were taken at 100 and 400x magnification (Olympus BX51 microscope, Olympus DP71 Digital Camera and DP manager software). Images for each gene were captured at the optimal exposure for each magnification. Images for Pri-miR132 were captured at the same exposure for both the 2 and 4 h time points. Quantification was performed on three individual animals across three slices each, spaced 80 μM apart. ImageJ (U.S. National Institutes of Health, Bethesda, MD, United States, http://imagej.nih.gov/ij/) was used to obtain the mean optical density (mean OD) of 400x images. The mean OD of the granule cell layer (GCL) or ML were compared to the region immediately below the ML (lateral posterior thalamic nucleus). The values for the stimulated and control side were then compared. The values were displayed as the fold change from the control side.

### NeuN IHC

Rats were perfused with 4% PFA in 1x PBS. The NeuN IHC was performed on free-floating coronal rat sections (40 μm). Sections were blocked in donkey serum in 1x PBS for 1 h at RT. They were then incubated overnight in an anti-NeuN antibody (1:500; Millipore #MAB377). Sections were washed with PBS for 10 min, 3x before being incubated with the secondary for 2 h at RT (1:200; Invitrogen #T6390). There were 3, 10 min final washes with PBS before being mounted onto superfrost slides (Fisher) and a fluorescent mounting media containing DAPI (Vectashield).

### Statistical Analysis

Statistics for the image quantification, microarray and qRT-PCR analysis data were done using two-tailed *t*-test assuming equal variances.

## Results

### Genome Wide DNA Microarray Screen Reveals Arc to Be the Most Prominent mRNA within the Molecular Layer Post-pp-HFS, Compared to Unstimulated Controls

**FIGURE 1:**
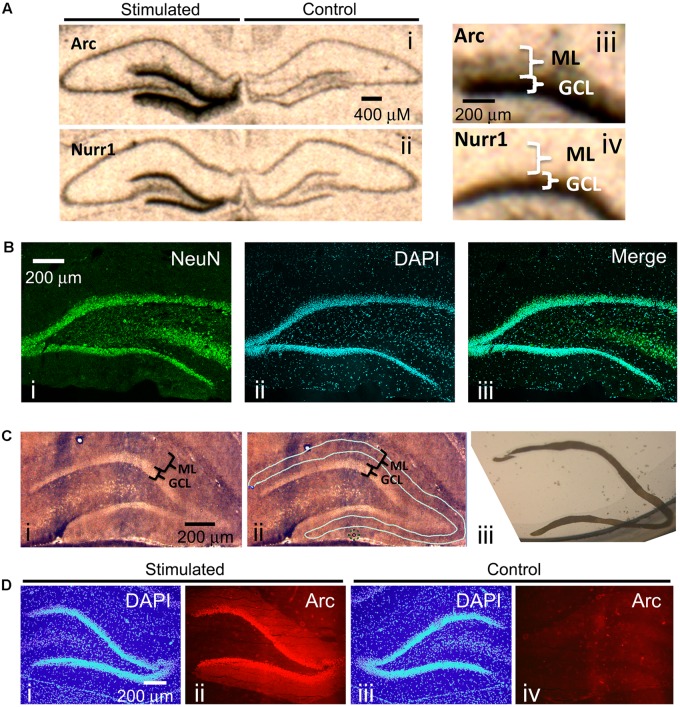
**(A)** Radioactive *In situ* hybridization of the immediate early genes (IEGs) Arc (i, iii) and Nurr1 (ii, iv) following unilateral pp-HFS to demonstrate robust presence of Arc mRNA in the ML 1 h following stimulation. Stimulated side indicates the side of the brain that received pp-HFS prior to sacrificing the rat. **(B)** A NeuN stain was performed to show the abundance of neuronal cell bodies in the GCL and their absence in the ML. A coronal section was stained for (i) NeuN and (ii) Dapi. (iii) A merge of NeuN and DAPI depicts the lack of NeuN positive cell bodies within the ML. **(C)** (i) A section containing the DG that was prepared for LMD. (ii) The same section is traced for the desired tissue sample of the ML prior to dissection. (iii) An image of the dissected ML tissue following LMD. **(D)** Confirmation of successful pp-HFS via Arc IHC. Staining of (i) DAPI and (ii) Arc protein on the stimulated side depicts robust localization of Arc to the ML compared to the contralateral/non-stimulated hemisphere (iii,iv), respectively.

One of the best methods for determining if specific mRNAs localize to dendrites is by *in situ* hybridization. *In situ* hybridization on hippocampal coronal tissue sections is especially ideal because of the unique neuroanatomical organization of the hippocampus: neuronal cell bodies are localized to discrete layers wherein the dendrites from these cell bodies project uniformly away from the soma and, therefore, spatially separate the cell body from the dendrites. These features of the hippocampus allowed for the serendipitous discovery that Arc mRNA localizes to dendrites (Link et al., 1995; Lyford et al., 1995) following neural activity of dentate gyrus neurons. Stimulating the angular bundle of the pp-HFS is one way to induce neural activity within the hippocampus, and specifically the dentate gyrus. This method is ideal because it utilizes a pattern of stimulation that produces highly reliable and robust alterations in synaptic plasticity (Bliss and Lomo, 1973; Malenka and Nicoll, 1999; Schafe et al., 2001; Rodrigues et al., 2004), and leads to robust gene expression within dentate gyrus granule cells. To illustrate this, we applied pp-HFS to urethane-anesthetized rats. One hour later, the brains were dissected and *in situ* hybridization was performed on coronal brain sections containing the dorsal hippocampus with radiolabeled ribo-probes for Arc mRNA and Nurr1 mRNA – two immediate early genes. As expected, both Arc mRNA and Nurr1 mRNA are robustly increased within the granule cell layer (GCL) of the dentate gyrus on the ipsilateral side of stimulation. In contrast, the contralateral side exhibits very low levels of these mRNAs. However, Arc mRNA is also detected in the molecular layer (ML) of the dentate gyrus on the ipsilateral side of stimulation, indicating that this mRNA is transported to the dendrites contained within the ML (**Figure 1A**). Notably, the ML consists of primarily dendrites, neuropil and traversing axons, but is virtually devoid of neuronal cell bodies. We performed immunohistochemistry for the neuronal marker NeuN, on coronal brain sections containing the dorsal hippocampus to demonstrate this. NeuN signal was very prominent in the neuronal cell bodies within the GCL of the dentate gyrus. However, there was a clear lack of NeuN stained cells within the ML (**Figure 1B**).

Next, we sought to identify all known transcripts within the rat dentate gyrus that are transported to the distal dendrites following synaptic activity induced by pp-HFS. Synaptic activity within the dentate gyrus of urethane-anesthetized rats induced by pp-HFS should lead to a robust increase in gene transcription of 100s of genes within GCL neurons, and we hypothesized that some of these mRNAs might be transported to the dendrites contained within the molecular layer of the dentate gyrus. These structures are so small, that accurate manual dissection would likely be impossible. Therefore, we utilized laser microdissection to dissect out the distal 2/3rds of the ML so we could ensure that we would avoid GCL contamination during the isolation of the ML (**Figure 1C**).

We stimulated the ipsilateral side (side of stimulation) with pp-HFS and then sacrificed the animals at 2 and 4 h post-stimulation. Cryocut coronal sections containing the anterior dorsal hippocampus were collected and subjected to Arc immunohistochemistry to verify that Arc gene expression was induced within the ipsilateral dentate gyrus (**Figure 1D**). We used this approach as an indirect way to verify the stimulation procedure was successful at inducing neural activity within the dentate gyrus for each animal that received pp-HFS. Next, additional cryocut sections were obtained from the dorsal hippocampus of animals that successfully received the pp-HFS. The ipsilateral and contralateral (unstimulated) distal 2/3rds of the molecular layer of the dentate gyrus was microdissected and the RNA was purified and amplified in preparation for DNA microarray analysis. We chose to analyze mRNA levels at 2 and 4 h post-stimulation compared to unstimulated controls because these are time points at which mRNAs would likely localize to dendrites following stimulation. For example, it has been previously demonstrated that Arc mRNA can be detected within the dendrites after only 30 min following stimulation and continues to accumulate within the dendrites for at least 2 h post-stimulation (Steward et al., 1998). During this period, numerous transcription factors are transcriptionally induced and promote transcription of a multitude of genes during additional waves of transcription. Two and four hours post-stimulation should be ideal time points to allow pp-HFS-induced genes to be transcribed and also allow enough time for dendrite-destined transcripts to accumulate within this compartment.

**FIGURE 2:**
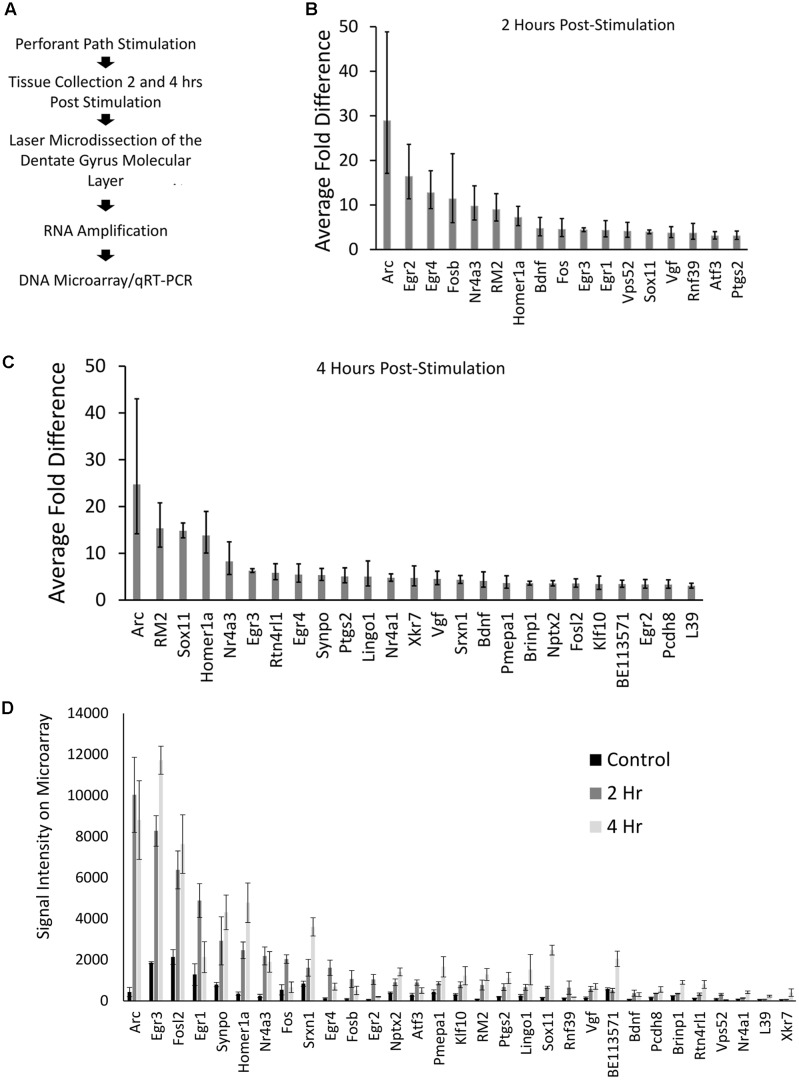
**(A)** Stepwise outline of experiment from surgery to DNA microarray. **(B)** Microarray results for 2 h post-pp-HFS, compared to controls, depicted as fold change. Error bars represent standard deviation (SD), *n* = 3 per group. **(C)** Microarray results for 4 h post pp-HFS, compared to controls, depicted as fold change. Error bars represent SD, *n* = 3 per group. **(D)** Raw signal intensity from DNA microarray for control, 2 h, and 4 h samples. Error bars represent standard error of the mean (SEM), *n* = 3 per group.

Our genome-wide screen, using Affymetrix DNA microarrays examined the expression levels of over 27,000 unique transcripts. After filtering data for changes of threefold or more, 31 transcripts were identified that were higher within the ML of the stimulated side (*p* < 0.05); one RNA exhibited a greater than threefold decrease in levels. Twenty-two of the 31 transcripts that were identified to be upregulated within the ML following pp-HFS, have been previously identified to be regulated via pp-HFS (Yamazaki et al., 2001; Matsuo et al., 2000; Ploski et al., 2010; Ryan et al., 2011, 2012). Eighteen of these 31 transcripts were also previously identified to be differentially regulated in neurons grown in culture following KCl-mediated induction of neural activity (Lin et al., 2008). The one transcript we identified to be decreased within the ML following pp-HFS (coiled-coil domain containing 177; Ccdc177), to our knowledge has not been previously identified to be regulated by pp-HFS, or KCl-mediated induction of neural activity. These types of studies have consistently identified a good number of genes that exhibit greater mRNA fold changes than Arc, so the fact that our screen identified Arc as the transcript with the highest increase in mRNA fold change within the ML 2 and 4 h following pp-HFS, is a good indication that the screen successfully identified mRNAs which have become enriched within the ML following pp-HFS (**Figure 2**). Annotated gene lists, including information regarding which genes were previously identified are provided in Supplementary File S1.

Another way to examine the microarray data is to view each gene’s signal intensity on the microarray comparing the control, 2 h, and 4 h samples (**Figure 2D**). Most of these genes exhibit fairly low signals on the microarray, which could indicate their overall levels are low in the ML. In contrast, Arc mRNA exhibits the highest signal intensity with only a few other mRNAs exhibiting similar levels.

**FIGURE 3:**
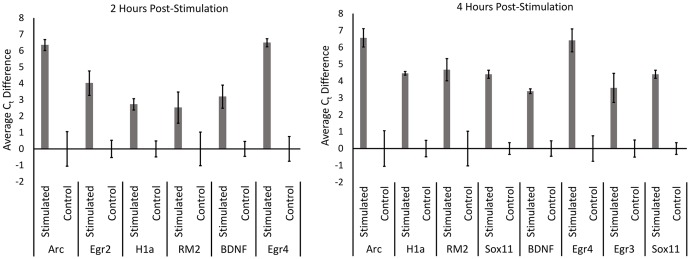
qRT-PCR was performed for control, 2 h, and 4 h samples from the microarray experiment. aRNA that was generated for the microarray was converted into cDNA and used to confirm results from the microarray via qRT-PCR. All genes tested exhibited significantly higher levels in stimulated samples compared to controls in both 2 and 4 h time points (*p* < 0.05) (with the exception of pri-miR132 2 h). Error bars represent SEM, *n* = 3 per group.

We next performed qRT-PCR for a subset of the identified candidate genes that either exhibited the greatest fold change or exhibited a strong signal on the microarray (**Figure 3**). For these experiments, we utilized the aRNA that was generated for the microarrays, converting it to cDNA and then performed qRT-PCR with gene specific primers. Each gene we examined exhibited significant increases in RNA levels compared to controls (with the exception of pri-miR132 at 2 h) (*n* = 3 per group; *p* < 0.05). Statistical data for these qRT-PCR experiments are provided in Supplementary File S3.

### RM2 Is a Non-coding Transcript Containing pri-miRNA132

**FIGURE 4:**
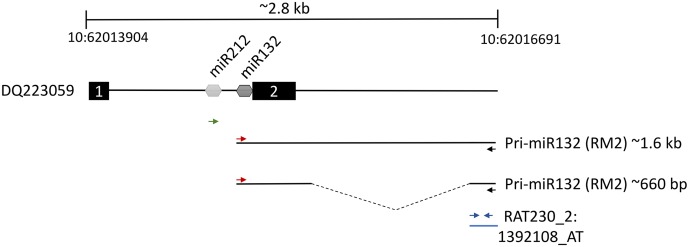
Two thousand eight hundred base pair region of rat chromosome 10 between nucleotides 62013904 and 62016691 containing the pri-miR132/RM2 gene loci. Dq223059 transcript contains both miR212 and miR132 between putative exon 1 and exon 2. We discovered a ∼1.6 kb and a ∼660 bp transcript that was present within ML samples that were collected 4 h post-pp-HFS. These transcripts contained the miR132 coding sequences to be contiguous with RNA sequences represented by the Affymetrix RM2 probe, RAT230_2:1392108_AT. The red and black arrows indicate the positions of the forward and reverse PCR primers, respectively, used to amplify the ∼1.6 kb and ∼660 bp transcripts. The green arrow represents the forward PCR primer intended to amplify the miR212 sequence when coupled with the RM2 reverse primer (black arrow); however, this primer pair did not yield a PCR product. Blue arrows indicate the locations of the PCR primers used for qRT-PCR of the pri-miR132/RM2 transcript. Dotted line within the ∼660 bp transcript indicates the location of a likely intron.

The second most robustly upregulated transcript at 4 h post-stimulation was a partially characterized transcript referred to as RM2 (GenBank: AB032083.1), which had reportedly been previously identified to be induced within the hippocampus following LTP inducing stimulation (Matsuo et al., 2000). There was no evidence that RM2 contained a reading frame to code for a protein. We reasoned that this transcript could be part of a larger, as of yet, unidentified transcript. Therefore, we examined the rat genome upstream from the RM2 genomic locus and we determined that a CREB dependent non-protein coding transcript that codes for the miRNAs, miRNA-212 and miRNA-132 (Vo et al., 2005) was directly adjacent to the RM2 locus. We hypothesized that the RM2 sequence might be part of a contiguous RNA that contains miRNA 212 and 132. To test this hypothesis, we subjected cDNA generated from RNA obtained from the distal molecular layer, 4 h post HFS (similar material that was used for the microarray analysis), to PCR with PCR primers specific for the RM2 microarray probe and the miRNA-212 or miRNA-132 loci. The PCR did not amplify a DNA product using the miRNA212 primer and RM2 primer, but it did amplify two PCR products that were ∼1.6 kb and ∼660 bp utilizing the miRNA132 and RM2 primers. These PCR products were cloned and subjected to DNA sequencing for verification. We determined that indeed RM2 was contiguous with an RNA transcript that contained miRNA132 (**Figure 4**). The ∼660 bp transcript was shorter than the ∼1.6 kb transcript because it was lacking a region intervening between miR132 and RM2, most likely due to the presence of an intron that was spliced out. These data are consistent with findings published while this work was ongoing (Takebayashi et al., 2014). Collectively, these findings are intriguing because miRNA132 is believed to localize to dendrites (Edbauer et al., 2010; Bicker et al., 2014), but it is currently unknown how it gets there (Tai and Schuman, 2006). Conventional wisdom dictates miRNAs are expressed within the nucleus as part of larger RNA polymerase II transcripts (pri-miRNAs), and these are cleaved within the nucleus by Drosha and DGCR8 to create pre-miRNAs, which are then exported to the cytoplasm for further processing by DICER to convert pre-miRNAs to mature miRNAs (Corbin et al., 2009; Bicker et al., 2014). However, our data indicate there is a large upregulation of pri-miRNA132/RM2 within the molecular layer (dendrites) following synaptic activity, indicating that pri-miR132 may leave the nucleus unprocessed. This possibility makes it plausible that miRNA132 is transported to the dendrites as a pri-miR132. Recent reports lend credibility to this model. For example, pri-miRNAs, Drosha and DGCR8 have recently been reported to be present within the post-synaptic density (PSD) (Lugli et al., 2012). These data indicate that miRNA132, might be transported to the dendrites following synaptic activity as a longer unprocessed transcript which is then likely processed to a mature miRNA when it reaches its destination. We will refer to RM2 as pri-miR132.

### Fluorescent *in Situ* Hybridization and qRT-PCR Indicate That Multiple Transcripts Localize to the ML Post-pp-HFS

**FIGURE 5:**
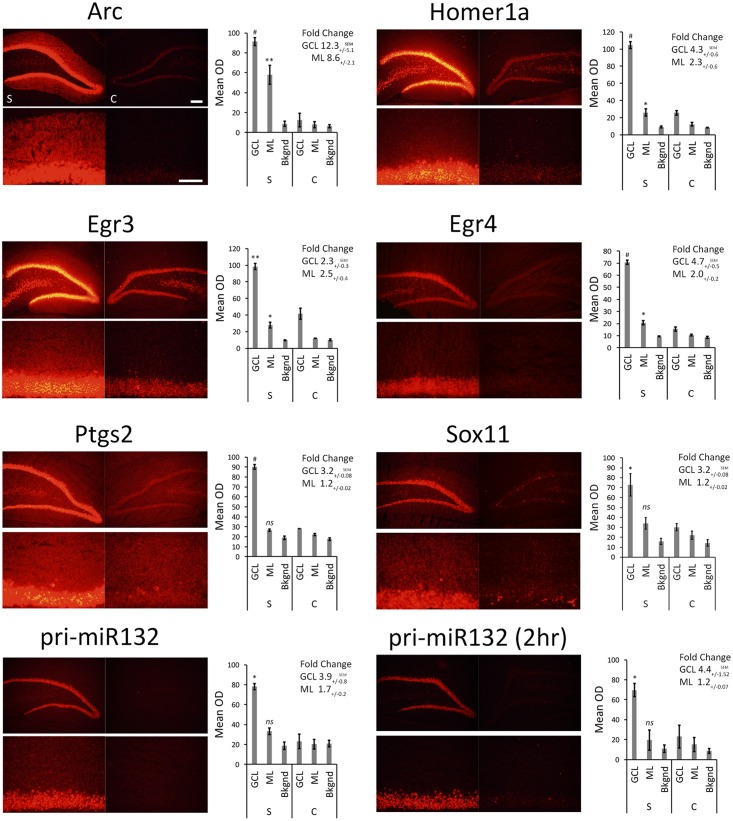
FISH for Arc, Egr3, Egr4, H1a, pri-miR132, Sox11, and Ptgs2. Rats were stimulated with pp-HFS and tissue was taken at the 4 h time point followed by FISH with transcript specific riboprobes. A 2 h time point is included for pri-miR132. Each slice was imaged at two different magnifications; zoom-out (top) and zoom-in (bottom). Control DG (right; c = control) and the stimulated DG (left; s = stimulated). The mean optical density of the *in situ* signal from the ML, GCL and background was quantified. (^∗^*p* < 0.05, ^∗∗^*p* < 0.005, ^#^*p* < 0.001) *ns*, not significant. Error bars represent SEM. *n* = 3 per group. Zoom-out Scale Bar: 200 um. Zoom-in Scale Bar: 100 um.

**FIGURE 6:**
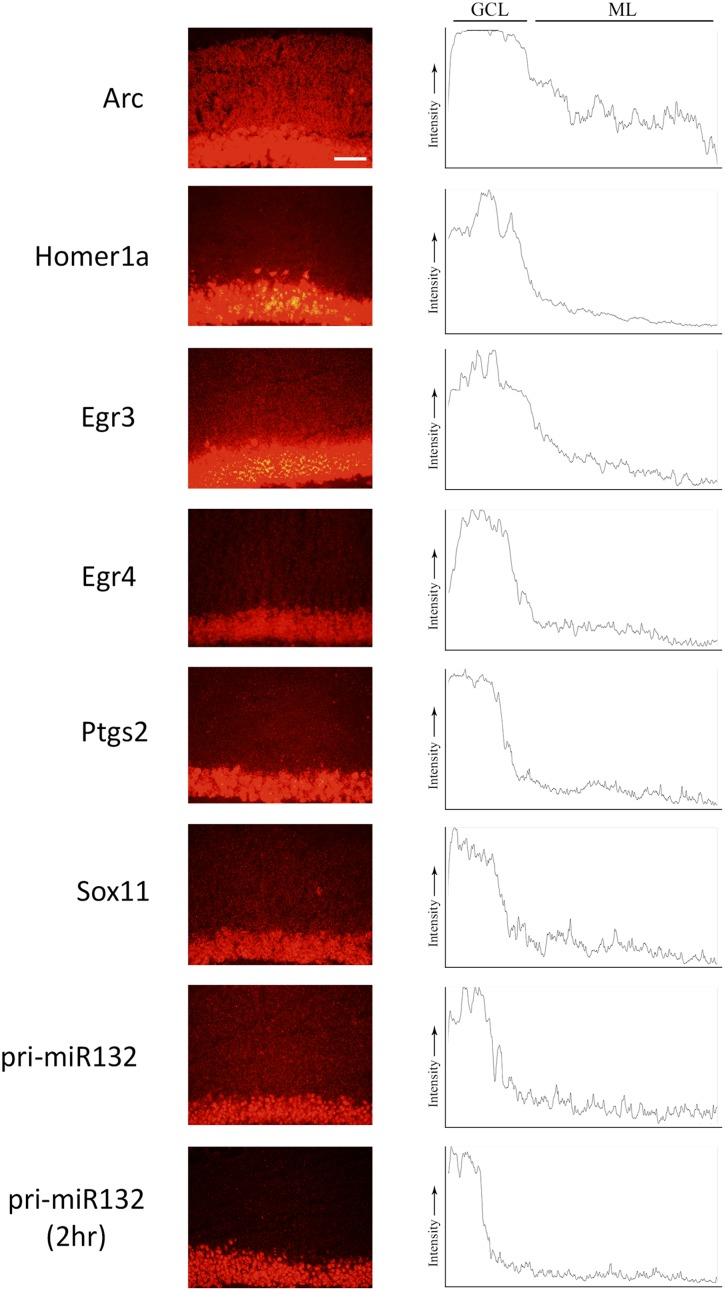
ImageJ was used to plot the intensity of the RNA assessed by FISH from the GCL and ML to visualize the signal across the two regions. Images were taken at the 4 h (and pri-miR132 2 h) timepoint at an optimal exposure for each gene. Plots are representative of the image displayed in this figure. Scale bar = 100 um.

We stimulated another group of animals with pp-HFS and 2 and 4 h later, the brains were dissected and coronal sections containing the dorsal hippocampus were taken. We performed secondary confirmation on a subset of candidate genes utilizing FISH using gene specific riboprobes (**Figure 5**). We chose to examine mRNA expression for the selected genes for the 4 h time point following pp-HFS, since this was the timepoint these genes exhibited the highest expression within the ML, according to the microarray data. For pri-miR132, we also examined expression at the 2 h time point. As expected, FISH performed for Arc yielded a robust and very convincing signal for Arc within the GCL and ML on the stimulated side of the brain. In contrast, the unstimulated, control side exhibited very little signal within these regions. We also examined the expression pattern for Egr3, Egr4, H1a, pri-miR132, Sox11, and Ptgs2 (*n* = 3). In all of these cases, these genes exhibited a significantly elevated expression signal within the GCL on the stimulated side of the brain as compared to the unstimulated hemisphere. Sox11, a known neurogenesis marker (Bergsland et al., 2006), is seen expressed in small subsets of cells within the subgranular zone within the GCL of the unstimulated side, but the stimulated side appears to express Sox11 in all of the GCL cells similar to the other mRNAs we examined. In contrast, there was generally little or no signal detected within the ML for these genes. Egr3, Egr4, and H1a exhibited a modest, but statistically significant signal within the ML on the stimulated side. These three mRNAs also appeared to be expressed within the hilus on the stimulated side. None of the genes appeared to be expressed by specific cells localized within the ML (i.e., glia). If the mRNAs for pri-miR132, Sox11, and Ptgs2 do indeed localize to dendrites within the ML, they likely do so at much lower levels than Arc mRNA, considering that our ability to detect them within the ML using FISH is limited. We also plotted the intensity of the FISH signal for these RNAs over the GCL and ML (**Figure 6**). Arc mRNA exhibits a robust GCL and ML signal. The other RNAs examined exhibit a much more modest ML signal as expected; however, these data indicate that Egr3 appears to extend further into the proximal dendrites. Statistical data for these FISH experiments are provided in Supplementary File S3.

**FIGURE 7:**
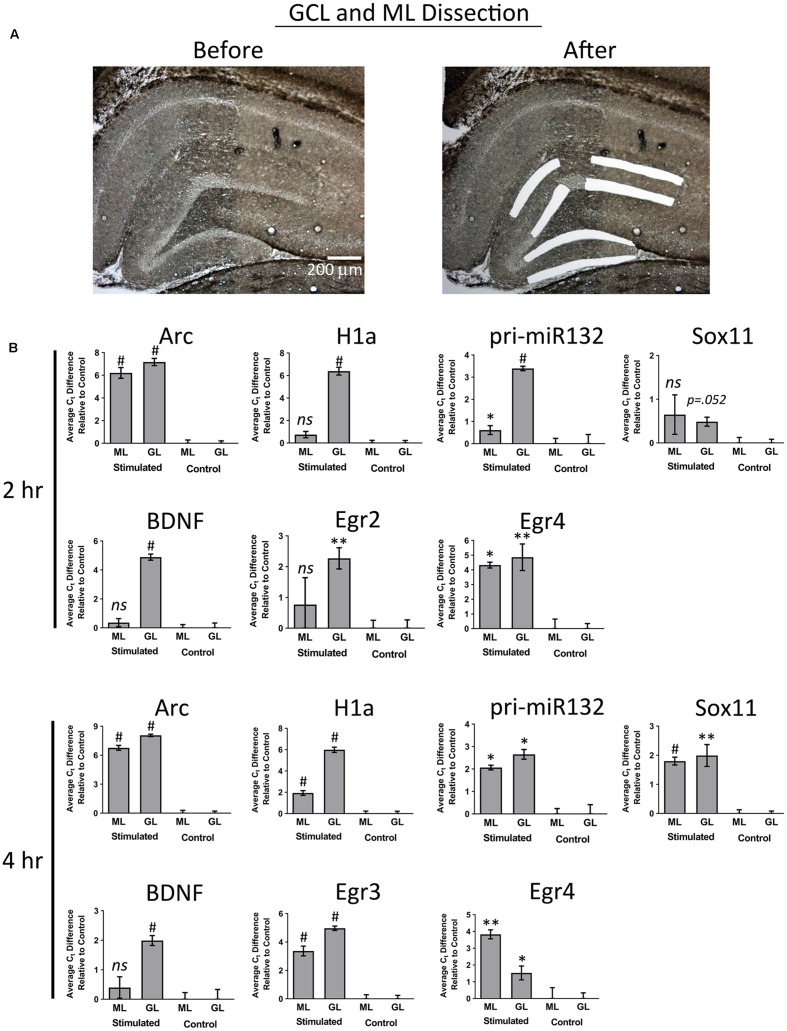
**(A)** Example images depicting tissue Before LMD and After LMD of GCL and ML tissue from coronal hippocampal sections. Note only the distal 1/3rd of the of the ML was dissected. **(B)** qRT-PCR was performed on these samples for Arc, H1a, pri-miR132, Sox11, BDNF, Egr2 (2 h only), Egr3 (4 h only) and Egr4, 2 h and 4 h post-pp-HFS. (^∗^*p* < 0.05, ^∗∗^*p* < 0.005, ^#^*p* < 0.001) *ns*, not significant. *n* = 5 for control, *n* = 4 for 2 h and 4 h samples. Error bars represent SEM.

In our final set of experiments, we sought to determine if utilizing a more sensitive approach could detect pp-HFS-induced mRNAs within the ML. We stimulated another group of animals with pp-HFS and then 2 and 4 h later, the brains were dissected and coronal sections containing the dorsal hippocampus were prepared for laser microdissection. This time we dissected tissue from the GCL from both the stimulated and unstimulated sides of the brain. We also dissected tissue from the distal most 1/3rd of the ML from both the stimulated and unstimulated sides of the brain. The RNA was isolated from this tissue and converted to cDNA and subsequently analyzed via qRT-PCR with gene specific primers (**Figure 7**). At 2 h post pp-HFS, Arc mRNA was detected in the GCL and ML on the stimulated sides compared to the unstimulated sides, as expected (*p* < 0.05; *n* = 4 for 2 h, *n* = 5 for control). H1a, pri-miR132, Sox11, BDNF, Egr2, and Egr4 exhibited an increase in the GCL on the stimulated side compared to the unstimulated side (*p* < 0.05; *n* = 4 for 2 h, *n* = 5 for control). However, for this time point, none of these targets exhibited an increase in the ML on the stimulated side compared to the unstimulated side (*p* > 0.05; *n* = 4 for 2 h, *n* = 5 for control), except for pri-miR132 and Egr4 (*p* < 0.05; *n* = 4 for 2 h, *n* = 5 for control). Notably, Egr4 exhibited a large increase in the ML, with a C_t_ difference of ∼4 or ∼16 fold. At 4 h post pp-HFS, Arc mRNA was detected in the GCL and ML on the stimulated sides compared to the unstimulated sides, as expected (*p* < 0.05; *n* = 4 for 4 h, *n* = 5 for control). H1a, pri-miR132, Sox11, BDNF, Egr3, and Egr4 exhibited an increase in the GCL on the stimulated side compared to the unstimulated side for this time point (*p* < 0.05; *n* = 4 for 4 h, *n* = 5 for control). The qRT-PCR also detected an increase for H1a, pri-miR132, Sox11, Egr3, and Egr4 within the ML on the stimulated side compared the unstimulated side for the 4 h time point (*p* < 0.05; *n* = 4 for 4 h, *n* = 5 for control). Interestingly, there wasn’t an appreciable increase in BDNF in the ML on the stimulated side compared to the non-stimulated side of the brain. Statistical data for these qRT-PCR experiments are provided in Supplementary File S3.

### Arc mRNA Is Relatively Abundant within the GCL and ML Compared to Other mRNAs Examined

**FIGURE 8:**
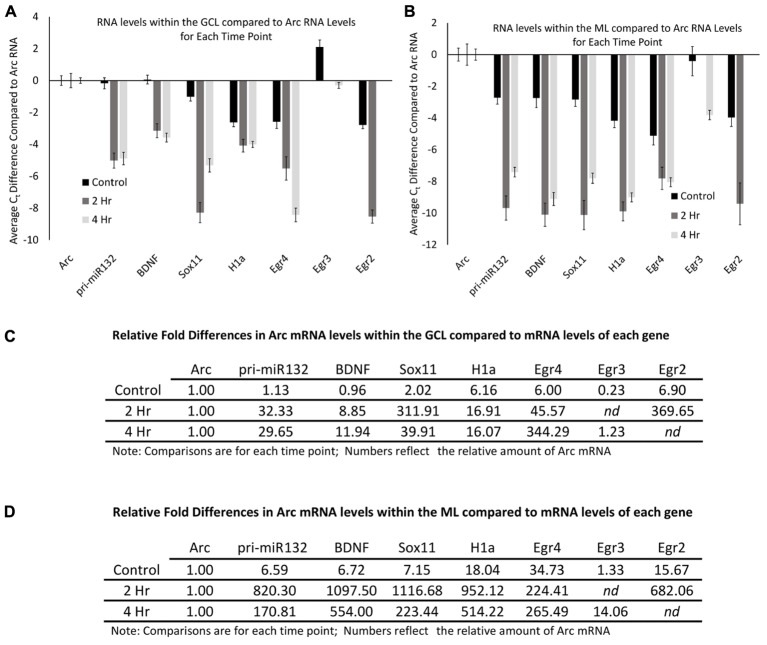
Arc mRNA levels at each time point (control, 2 h, 4 h), are much higher than other candidate gene transcripts examined. **(A)** mRNA levels in reference to Arc mRNA levels within the GCL. Comparisons are between each mRNA examined compared to Arc mRNA for each time point. Negative C_t_ values indicate lower mRNA levels. (*n* = 5 for control, *n* = 4 for 2 and 4 h samples). Error bars represent SEM. **(B)** Comparison of mRNA levels in reference to Arc mRNA levels within the ML. Comparisons are between each mRNA examined compared to Arc mRNA for each time point. Negative C_t_ values indicate lower mRNA levels (*n* = 5 for control, n = 4 for 2 and 4 h samples). Error bars represent SEM. **(C)** Relative fold differences of Arc compared to the other mRNAs examined for each time point within the GCL. Numbers reflect the relative amount of Arc mRNA. In most comparisons presented, Arc exhibits higher levels of mRNA than the other genes analyzed at each time point. Numbers above one indicates higher levels of Arc mRNA. *nd*, not determined. **(D)** Relative fold differences of Arc compared to the other mRNAs examined for each time point within the ML. Numbers reflect the relative amount of Arc mRNA. Numbers above one indicates higher levels of Arc mRNA. In most comparisons presented, Arc exhibits higher levels of mRNA than the other genes analyzed at each time point. *nd*, not determined.

**FIGURE 9:**
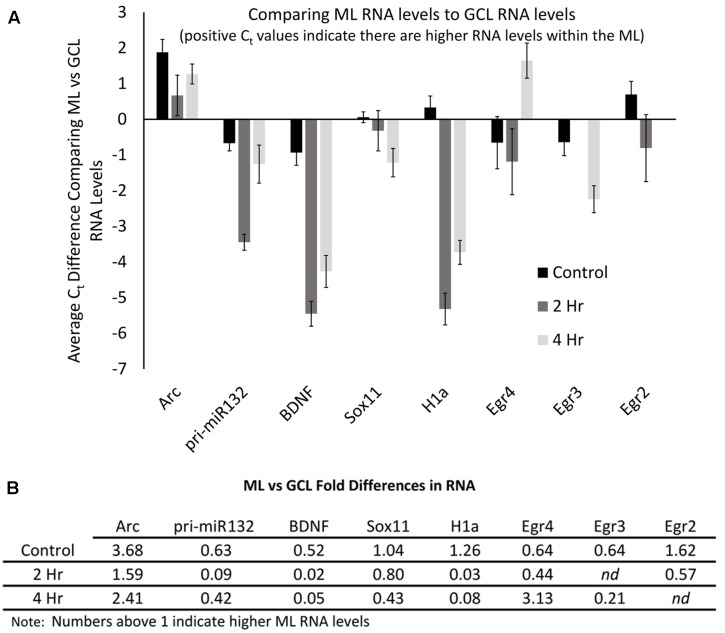
Arc mRNA levels are proportionally higher within the ML compared to the GCL. **(A)** Levels of each mRNA in the ML compared to their levels in the GCL for each time point. Positive C_t_ values indicate higher levels within the ML. (*n* = 5 for control, *n* = 4 for 2 and 4 h samples). Error bars represent SEM. **(B)** Fold differences of RNA levels in the ML, compared to the GCL for each time point. Numbers above 1 indicate higher mRNA levels within the ML (*n* = 5 for control, *n* = 4 for 2 and 4 h samples). *nd*, not determined.

The qRT-PCR data allowed us to examine the relative levels of these mRNAs within the dentate gyrus, which could provide some insight into how these differing mRNAs compare to the levels of Arc mRNA. To do this, we first normalized the levels of each RNA to the levels of GAPDH mRNA. We then compared the control GCL sample Arc mRNA levels to the other control GCL mRNA samples examined (i.e., Egr2, Egr3, etc.). We found that Arc mRNA levels were modestly higher than the other mRNAs examined except for Egr3 (**Figure 8A**); however, when we compared Arc GCL mRNA levels at the 2 and 4 h timepoints to the other GCL mRNAs examined at these time points, we found that Arc levels were much higher than the other mRNAs for these time points. We then compared the control sample’s Arc mRNA levels within the ML to the other control ML mRNAs. We found that Arc ML mRNA levels were higher than the other ML mRNAs examined (**Figure 8B**). When we compared the 2 and 4 h timepoint Arc mRNA levels within the ML to the other mRNAs examined for the 2 and 4 h time points, we found that Arc ML levels were significantly higher than the other mRNAs. We report the relative fold differences of Arc mRNA compared to the other mRNAs examined in the GCL and ML (**Figures 8C,D**). Arc mRNA generally is more abundant than the other mRNAs examined. Within the ML compartment, Arc levels can exceed the levels of some of these other mRNAs 100–1000-fold. In our last analysis, we compared the GCL versus ML levels of each mRNA (**Figures 9A,B**). We found that Arc mRNA levels were consistently higher within the ML compared to the GCL across all samples (control, 2 h, and 4 h). In contrast, virtually all the other mRNAs examined exhibited much higher GCL levels compared to ML levels. An important exception was Egr4 at the 4 h time point. Collectively, these mRNA level findings corroborate the DNA microarray findings indicating that Arc mRNA levels are much higher within the ML compared to most of the other mRNAs we examined. Additionally, these data support the notion that while some of these mRNAs do increase their levels within the ML following pp-HFS, their relative levels compared to Arc mRNA are exceedingly low and this is likely the main reason why FISH analysis was unable to convincingly detect these candidate mRNAs within the ML.

## Discussion

Decades ago, Arc was serendipitously identified to localize to dendrites of granule cell neurons within the hippocampus following neural activity (Link et al., 1995; Lyford et al., 1995). Besides this being an intriguing phenomenon, arguably, the most noteworthy aspect of this phenomenon was how robust it is and how easy it is to detect using *in situ* hybridization. To date, no other activity dependent transcript has been shown with such unambiguity to localize to dendrites. Considering this fact, one may wonder if Arc mRNA is unique among activity dependent transcripts for its ability to accumulate within the dendrites with such robustness and efficiency. Because of this, we sought to identify all known transcripts within the GCL of the rat dentate gyrus which are transported to the distal dendrites following synaptic activity induced by pp-HFS. Our genome wide screen revealed that out of 27,000 unique transcripts represented on the Affymetrix DNA microarray, Arc exhibited the greatest accumulation within the dendrites following pp-HFS. Subsequent experiments utilizing FISH and qRT-PCR generated data consistent with this observation. Our study also revealed other activity dependent transcripts likely localize to granule cell dendrites following pp-HFS, but do so with much less robustness and efficiency. In particular, we report that Homer1A, Egr3, and Egr4 transcripts localize to the dendrites of granule cell neurons following pp-HFS. We also identified pri-miR132 RNA as being localized to granule cell dendrites; however, our FISH experiments were not able to confirm this finding, and this may be due technical limitations of the FISH technique not being capable of detecting low levels of this transcript. Additional analysis of the qRT-PCR data revealed that Arc is generally a much more abundant mRNA within the GCL and ML compared to the other RNAs examined and it possesses proportionally more mRNA within the ML compared to the GCL for all samples tested (i.e., control, 2 h, and 4 h). Arc mRNA appears to be a very abundant activity dependent transcript, and it is this abundance that likely contributes to the ability to detect Arc mRNA within the dendrites of the ML with impressive ease and unambiguity when utilizing FISH.

The design of our study benefits from the fact that pp-HFS leads to robust induction of gene expression within the dentate gyrus and associated molecular layer and this dramatically increases the signal to noise ratio that is required for optimal gene discovery. Additionally, the dentate gyrus ML can be easily identified allowing for highly accurate laser microdissection, eliminating gene dilution/negation effects due to contamination from surrounding structures, such as the GCL. Furthermore, the use of the *in vivo* anesthetized LTP preparation has the additional advantage of avoiding gene dilution effects which may be inherent in either awake-behaving models, due to variability in the baseline expression of activity-dependent genes (Cirelli et al., 2004), or *in vitro* slice methods, in which cutting the brain slice alone may result in significant changes in gene expression (Taubenfeld et al., 2002). So, in theory our study design should provide a unique method to detect transcripts that accumulate in the dendrites contained within the ML following pp-HFS, with a few caveats. The ML is composed primarily of dendrites from GCL neurons, but glial cells are present too. So, it is a possibility that some of the 31 genes we identified in our microarray screen to be increased within the ML following pp-HFS could be due to an increase in expression within glial cells contained within the ML. However, for this to be the case it would mean that pp-HFS would have had to induce ML glial gene expression on the side of stimulation and glial cells contained within the ML of the unstimulated side did not exhibit a commensurate increase in gene expression. While we cannot rule out this possibility for every candidate gene we identified, there are a number of reasons this would be unlikely and/or would not significantly influence our main findings.

Most of the transcripts we identified, have previously been identified to be increased due to pp-HFS or KCl-mediated neuronal depolarization (Matsuo et al., 2000; Yamazaki et al., 2001; Ploski et al., 2010; Ryan et al., 2011, 2012) and are; therefore, generally believed to be of neuronal origin. While this doesn’t rule out that the expression could be also originating from glial cells, *in situ* hybridization experiments from our study and others (Ploski et al., 2010), have determined that expression of many of these genes are clearly of neuronal origin and, if there is glial expression, is it below the detectable level utilizing *in situ* hybridization. For example, if gene expression within glia was changing due to pp-HFS, we should have detected it within cellular nuclei within the ML following pp-HFS during our FISH experiments. Instead, every gene that we examined for gene expression using FISH following pp-HFS, exhibited a clear GCL signal indicating neuronal origin and there was no indication that gene expression was being induced within specific cellular nuclei within the ML, indicating that expression from glial cells was an unlikely source for the origin of these transcripts. FISH revealed that Egr3, Homer1A, and Egr4 exhibited a diffuse signal within the confines of the ML on the stimulated side of the brain, consistent with dendritic localization of these transcripts. The induction of glial gene expression within the ML due to pp-HFS has never been reported, but even if some of transcripts that we identified in our screen are of glial origin, the fact remains that there is no evidence of any other activity dependent transcript being comparable to Arc, in its ability to locate to the dendrites with such efficiency or magnitude.

It also remains possible that our microarray screen might have identified transcripts that increase their levels within the ML due to pp-HFS, that are not activity dependent transcripts. For example some transcripts such as alpha Ca^2+^/calmodulin-dependent protein kinases II (αCaMKII), are constitutively expressed and localize to the dendrites, but synaptic activity has been shown to enhance their trafficking to the dendrites (Mori et al., 2000; Havik et al., 2003). We did not identify αCaMKII transcripts in our microarray screen, quite possibly due to the fact that the enhanced trafficking that αCaMKII might have undergone, was not significant enough to reach the threshold set by our filtering of the microarray data. Most of the genes identified in our screen have been previously identified to be activity dependent genes (Lin et al., 2008; Ploski et al., 2010).

In our study, we chose to analyze mRNA levels at 2 and 4 h post-stimulation compared to unstimulated controls utilizing DNA microarray technology; however, we could have utilized RNAseq technology instead. We opted to utilize DNA microarrays in part because we suspected that the transcripts of interest (i.e., RNAs that localize to dendrites following pp-HFS), would likely be relatively rare transcripts compared to the total cellular RNAs that might be present within the ML. Since DNA microarray is a probe based technology, by design, as long as the transcripts for a particular probe produce a signal above a threshold level, we would be able to collect data regarding the specific transcript/gene, and easily filter out all of the genes that did not differ in their expression between the stimulated and unstimulated sides. In contrast, RNAseq relies on the chance event of sequencing each transcript within the sample; therefore, transcripts of low abundance might never be sequenced and therefore no data would be collected regarding those transcripts. This issue can be overcome by increasing the sequencing depth for each sample. Since next generation DNA sequencing costs have dropped dramatically over the years, this is less of a concern, since it is no longer cost prohibitive to perform extensive next generation sequencing to ensure an adequate sequencing depth. When we performed the DNA microarray experiment; however, we opted to take a more cautious approach as it remained uncertain what sequencing depth would be required to adequately analyze our samples.

The time periods we examined RNA expression were based on three factors: the time period pp-HFS induced gene expression would likely occur, the amount of time it might take for transcribed RNA to accumulate within the dendrites, and keeping the number of samples within a manageable range. Previous studies have indicated that Arc mRNA is transcribed relatively rapidly following pp-HFS and it accumulates within the dendrites over many minutes and hours, where 2 h post-pp-HFS appears to have a stronger Arc mRNA signal within the dendrites compared to earlier time points (Steward et al., 1998). Based on this phenomenon, we reasoned that if there were other immediate early genes expressed due to pp-HFS and transported to the dendrites, 2 h pp-HFS would likely be a good time point to capture them. Because pp-HFS also induces the expression of numerous transcription factors that create additional waves of transcription, if some of these transcripts are transported to the dendrites, we thought we might be able to capture them at the 4 h time point, when they might have had enough time to accumulate within the dendrites. However, the fact remains that we might have missed transcripts if they had a relatively short half-life within the dendrites, or if their accumulation within the dendrites didn’t occur until after the 4 h time point. Interestingly, Arc mRNA expression has been shown to express for up to 8 h within GCL neurons due to exploratory behavior indicating that gene expression might continue on for hours post-stimulation (Ramirez-Amaya et al., 2005). Consistent with this view, one study identified gene expression changes 5 and 24 h after pp-HFS, but in most cases the gene expression changes were modest at these time points indicating the robust transcription changes seen shortly after pp-HFS were ending or over (Ryan et al., 2012).

Numerous attempts have been made to identify the mRNAs that localize to dendrites. Some of these attempts have used a candidate gene approach, while others have utilized an unbiased screening approach. For example, some studies have isolated mRNA selectively from dendrites by dissecting out portions of dendrites from sparsely populated neuronal cultures followed by RNA amplification to generate enough RNA suitable for differential display or DNA microarray screens (Miyashiro et al., 1994; Eberwine et al., 2002). One study used a similar approach but grew the neurons on a porous filter that allowed neuronal processes to pass through the pores of the filter to physically separate the neuronal processes from the cell bodies. The neuronal processes were then mechanically sheared from the neuronal cells bodies, the RNA was isolated, amplified and subjected to DNA microarray, resulting in many candidate genes (Poon et al., 2006). Other notable approaches for identifying mRNAs which localize to dendrites have included biochemical fractionation of synaptoneurosomes (Sung et al., 2004) or PSD (Tian et al., 1999) fractions followed by the isolation and examination of the RNA contained within these fractions. RNA has been isolated from manually dissected dendrites and cell bodies from the CA1 region of the hippocampus followed by DNA microarray analysis to compare differential abundance of RNAs between these two regions (Zhong et al., 2006). A more recent study (Cajigas et al., 2012) manually dissected dendrites and cell bodies from the CA1 region of the hippocampus and subjected normalized cDNA libraries generated from the dissected material to 454 Deep Sequencing and identified over 8000 genes (∼ a third of the genome). The authors of this study then used bioinformatics to remove genes that might have been detected due to contamination from glia, cell bodies and interneurons to reduce the list to ∼ 2500 genes. In the discussion of this paper the authors claim that half of all genes expressed within CA1 neurons contain their mRNAs within the dendrites of CA1 neurons. Similar studies have been conducted to detect micro-RNAs (miRNAs) within dendrites (Kye et al., 2007; Lugli et al., 2008; Siegel et al., 2009). Collectively, all of these studies are technically demanding and noteworthy; however, there is little consistency among the genes identified between these above-mentioned studies and some are lacking specific information regarding which transcripts were identified due to a lack of bioinformatic information available at the time these studies were conducted.

Our data indicate multiple genes might increase their mRNAs within the distal dendrites of the ML following pp-HFS; however, these genes do so at much lower levels than Arc. Some of these mRNAs code for transcription factors (Sox11, Egr2, Egr3, and Egr4). This is an is intriguing finding, but it is of course difficult to explain why nuclear localized proteins would have their mRNAs present within the dendrites. One possibility is that these transcription factors are locally translated following synaptic activity and then the proteins localize to the nucleus to regulate gene expression. This model provides an elegant mechanism for synapse to nucleus signaling. However, one obvious flaw with this view, is that our data indicate these RNAs localize to dendrites hours after the synaptic activity and it remains uncertain if they would remain within this compartment long enough to effectively serve as a link between future synaptic activity and the nucleus. Alternatively, maybe most of these RNAs are present within the dendrites simply due to diffusion and there is no physiological significance for their existence within the dendrites. For example findings from Cajigas et al. (2012), indicate that many RNAs from many different genes (possibly 1000s), have RNAs that localize to the dendrites. We suspect that in many of these cases there likely isn’t a physiological reason why these RNAs are within the dendrites. Notably, even for highly studied mRNAs like Arc, where high levels of Arc mRNAs are present within the dendrites of the ML, it still remains undetermined what the true significance of localizing Arc mRNA to the dendrites is, because no such studies have been performed to answer this question. It is generally accepted that Arc mRNA is locally translated at activated synapses. But would there be a physiological consequence if Arc mRNA was restricted to the cell body region? More research is needed to determine this.

## Conclusion

Our data indicate Arc is a unique activity dependent gene, due to the magnitude and efficiency which its activity dependent transcript localizes to the dendrites. Our study determined other activity dependent transcripts likely localize their transcripts to the dendrites following neural activity, but do so with significantly less magnitude compared to Arc.

## Author Contributions

CdS, AM, MH, and AP performed the dissection of the tissue for this study. CdS and AP performed *in situ* hybridization and fluorescent *in situ* hybridization and associated quantitation. CdS purified and amplified RNA of all samples in preparation for the microarray and qRT-PCR. CdS performed the IHC. JP and CdS designed the original experiments, analyzed all data and drafted the manuscript. All authors read, edited, and approved the final manuscript.

## Conflict of Interest Statement

The authors declare that the research was conducted in the absence of any commercial or financial relationships that could be construed as a potential conflict of interest.
