# Supplementary material for: Is Arc mRNA Unique: A Search for mRNAs That Localize to the Distal Dendrites of Dentate Gyrus Granule Cells Following Neural Activity
Source: Front Mol Neurosci. 2017 Oct 10;10:314. doi: 10.3389/fnmol.2017.00314 (PMC5641362; doi:10.3389/fnmol.2017.00314)
Supplement: Supplementary file 2 [file Data_Sheet_2.PDF]

A.

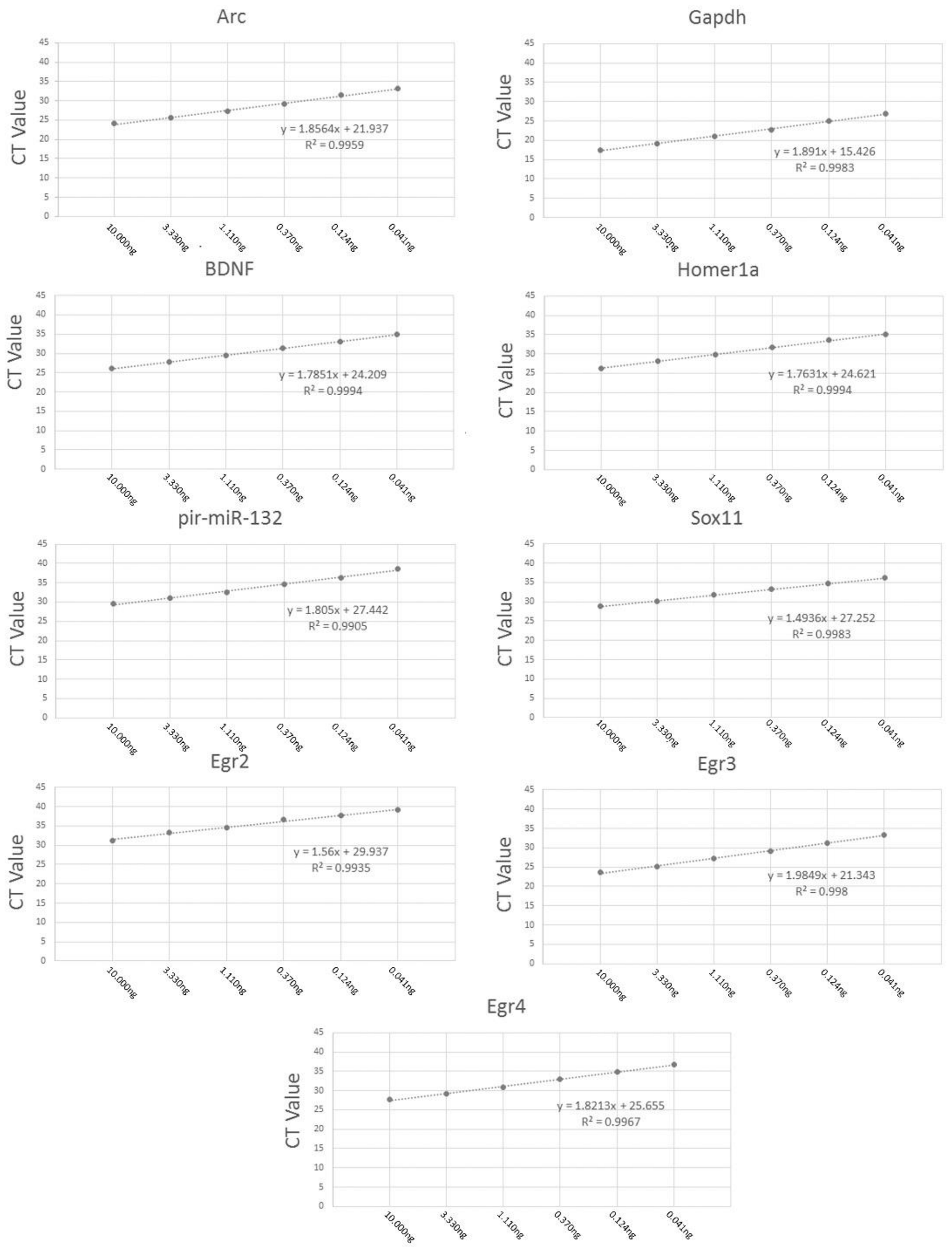

Supplemental Figure 2 (S2.A): Confirmation of qRT-PCR primers. A defined amount of RNA, extracted from rat hippocampus, was converted to cDNA using Superscript Reverse Transcriptase ll. Six samples, in duplicate were subjected to qRT-PCR, each a 1:3 dilution of the previous sample (10ng, 3.33ng, 1.11ng, 0.037ng, 0.124ng and 0.041ng). The scatter plot, slope and R^2 are provided for each gene.

B.

Primers used for qRTPCR

Arc	FP	CCCTGCAGCCCAAGTTCAAG
	RP	GAAGGCTCGCTGCCTGCTC
pri-miR-132	FP	TCCTGGCACCAGAAATAAACG
	RP	ACAAAAGCATGCCCCAGCAC
BDNF	FP	AAGGCTGCAGGGGCATAGAC
	RP	TGAACCGCCAGCCAATTCTC
Sox11	FP	CTCCTCGGGAGGCAGTCG
	RP	TCTGCGCCACATCTCTGACC
Homer1a	FP	CTGCTCCAAAGGAAAGCCTTGC
	RP	AAACAACCTTCAATGCTGACGG
Egr2	FP	GAAGCGCCACACCAAGATCC
	RP	CCTCCAATGGCGCTGTTACC
Egr3	FP	GCGCTCAGTACGCAGACGAC
	RP	GTCGCCGCAGTTGGAATAGG
Egr4	FP	CTGCCCCGTGGAGAGCTG
	RP	TGAAGTTGCGCAGGCAGATG
Gapdh	FP	GCATCCTGCACCACCAACTG
	RP	ACGCCACAGCTTTCCAGAGG

Primers used to generate in situ Probes

pri-miR132	FP	CAGGGCAACCGTGGCTTTCGATTGTTACTGTGGGAACCGG
	RP	GGTCTCACTGTAGTTCTGGCTAGCCTTGAACTCACAGAAACCC
Sox11	FP	CTCCTCTGAGCTGCTCGATC
	RP	CGGCTTGGCAAACAAAGCCTTAC
Egr3	FP	CCTCGAGATGACCGGCAAACTCGCCGAG
	RP	AATACGACTCACTATAGGGAGAGGGCGCAGGTGGTGACCACAGG
Cox2	FP	CGCTCAGCCATGCAGCAAATCC
	RP	GGGTTAATGTCATCTAGTCTGGAGTGGG
Egr4	FP	CCTCGAGATGCTCCACCTGAGCGACTTC
	RP	CAGCGCGGCGAAAGAGAGGCCCAGC

Supplemental Figure 2 (S2.B): A list of primers used for qRT-PCR and primers used to amplify sequences for *in situ* probe templates.
