# Supplementary material for: Is Arc mRNA Unique: A Search for mRNAs That Localize to the Distal Dendrites of Dentate Gyrus Granule Cells Following Neural Activity
Source: Front Mol Neurosci. 2017 Oct 10;10:314. doi: 10.3389/fnmol.2017.00314 (PMC5641362; doi:10.3389/fnmol.2017.00314)
Supplement: Supplementary file 3 [file Data_Sheet_3.PDF]

S3. Figure 3, 5 and 6 Statistics

qRTPCR of Microarray Samples (Figure 3)

		p	Tstat
2 Hr	Arc	0.005235384	5.527
4 Hr	Arc	0.006010528	5.319
2 Hr	BDNF	0.019112599	3.799
4 Hr	BDNF	0.002055996	7.121
2 Hr	Homer1a	0.010397923	4.552
4 Hr	Homer1a	0.000865502	8.941
2 Hr	Egr3	0.011763453	4.392
4 Hr	Egr3	0.022871237	3.594
2 Hr	Egr4	0.001226428	8.162
4 Hr	Egr4	0.003228142	6.309
4 Hr	Sox11	0.000473963	10.449
2 Hr	pri-miR132	0.146500796	1.798
4 Hr	pri-miR132	0.018554421	3.834

Control n=3; 4 Hr n=3; 2 Hr n=3

in situ Quantification (Figure 5)
GCL: Stimualted vs Control

	p	Tstat
Arc	0.0004622	10.517
Homer1a	0.0000499	18.530
Egr3	0.0018237	7.351
Egr4	0.0000150	25.099
Ptgs2	0.0000026	38.827
Sox11	0.0119573	4.371
pri-miR132	0.0055121	5.448
pri-miR132 (2 Hr)	0.0092211	4.713

Control n=3; Stimulated n=3

in situ Quantification (Figure 5)
ML: Stimualted vs Control

	p	Tstat
Arc	0.0025699	6.708
Homer1a	0.0418832	2.952
Egr3	0.0088150	4.774
Egr4	0.0075926	4.981
Ptgs2	0.1199903	1.971
Sox11	0.47322114	0.791
pri-miR132	0.1765127	1.639
pri-miR132 (2 Hr)	0.7825205	0.295

Control n=3; Stimulated n=3

qRTPCR of GCL and ML (Figure 6)
ML: Stimulated vs Control

		p	Tstat
2 Hr	Arc	0.0000854	11.724
4 Hr	Arc	0.0000005	17.096
2 Hr	BDNF	0.4270680	0.815
4 Hr	BDNF	0.3672717	0.922
2 Hr	Homer1a	0.0871780	2.038
4 Hr	Homer1a	0.0006023	5.809
2 Hr	Egr2	0.4532284	0.934
4 Hr	Egr2	*nd*	*nd*
2 Hr	Egr3	*nd*	*nd*
4 Hr	Egr3	0.0002308	7.550
2 Hr	Egr4	0.0017489	5.713
4 Hr	Egr4	0.0023898	4.949
2 Hr	Sox11	0.2493412	1.536
4 Hr	Sox11	0.0000322	9.693
2 Hr	pri-miR132	0.0458440	3.004
4 Hr	pri-miR132	0.0024867	8.031

Control n=5; 4 Hr n=4; 2 Hr n=4

qRTPCR of GCL and ML (Figure 6)
GCL: Stimulated vs Control

		p	Tstat
2 Hr	Arc	0.0000027	20.057
4 Hr	Arc	0.0000001	27.586
2 Hr	BDNF	0.0000024	19.356
4 Hr	BDNF	0.0007550	10.134
2 Hr	Homer1a	0.0000118	16.139
4 Hr	Homer1a	0.0000008	17.793
2 Hr	Egr2	0.0019791	5.277
4 Hr	Egr2	*nd*	*nd*
2 Hr	Egr3	*nd*	*nd*
4 Hr	Egr3	0.0000013	16.066
2 Hr	Egr4	0.0080544	5.506
4 Hr	Egr4	0.0273757	2.887
2 Hr	Sox11	0.0526621	3.282
4 Hr	Sox11	0.0028880	8.948
2 Hr	pri-miR132	0.0000056	14.265
4 Hr	pri-miR132	0.0058658	5.741

Control n=5; 4 Hr n=4; 2 Hr n=4

qRTPCR of GCL and ML (Figure 6)
ML: 2 Hr vs 4 Hr

	p	Tstat
Arc	0.3542154	1.030
BDNF	0.9259294	0.097
Homer1a	0.0175692	3.302
Egr2	*nd*	*nd*
Egr3	*nd*	*nd*
Egr4	0.1937313	1.480
Sox11	0.0798914	2.440
pri-miR132	0.0057621	4.502

Control n=5; 4 Hr n=4; 2 Hr n=4

qRTPCR of GCL and ML (Figure 6)
GCL: 2 Hr vs 4 Hr

	p	Tstat
Arc	0.8903248	0.147
BDNF	0.0453306	2.694
Homer1a	0.3868788	0.940
Egr2	*nd*	*nd*
Egr3	*nd*	*nd*
Egr4	0.0264433	3.354
Sox11	0.0069674	4.037
pri-miR132	0.2083755	1.562

Control n=5; 4 Hr n=4; 2 Hr n=4

qRTPCR of GCL and ML (Figure 6)
GCL vs ML

		p	Tstat			*p*	Tstat
2 Hr	Arc	0.2917397	1.172	4 Hr	Egr3	0.0036289	5.927
4 Hr	Arc	0.0086008	4.530	2 Hr	Egr4	0.2813487	1.287
2 Hr	BDNF	0.0000076	15.651	4 Hr	Egr4	0.0195007	3.343
4 Hr	BDNF	0.0001623	9.517	2 Hr	Sox11	0.5916628	0.569
2 Hr	Homer1a	0.0000278	11.942	4 Hr	Sox11	0.0414128	3.050
4 Hr	Homer1a	0.0000349	11.083	2 Hr	pri-miR132	0.0000503	12.658
2 Hr	Egr2	0.4385327	0.861	4 Hr	pri-miR132	0.0673866	2.353

Control n=5; 4 Hr n=4; 2 Hr n=4

Supplemental File 3 (S3.). P-values and T statistic (Tstat) for all statistics presented in figures 3, 5 and 6. All statistics were done using a Two-sample, Two-tailed t-test assuming equal variances, where a *p* of <0.05 is considered significant. *nd* = not determined.
